# VALUE-BASED HEALTHCARE AS A FRAMEWORK OF CARE FOR OLDER ADULTS AND THEIR FAMILY CAREGIVERS

**DOI:** 10.1093/geroni/igz038.498

**Published:** 2019-11-08

**Authors:** Gwen McGhan, Fiona Clement, Natalie Ludlow, Jessica Lee, Michelle Cheng, Deirdre McCaughey

**Affiliations:** University of Calgary, Calgary, Alberta, Canada

## Abstract

Value-based healthcare (VBHC) is a term synonymous with the pursuit of greater value in healthcare, however, the term “value” lacks conceptual clarity. Using Porter’s 2010 seminal VBHC shows promise for placing the older adult and their family caregiver at the center of care models, which is a central tenet in healthcare value. The purpose of our study is to conceptualize how Porter’s VBHC is defined, operationalized, and implemented to address the need for person-and-family centered care models that improve outcomes while being cost effective. A literature search in six academic databases was conducted to identify articles examining VBHC; specifically studies with a Porter-based patient-centric focus. 1,001 articles were retrieved for initial review. Using a consensus-based logic model for inclusion/exclusion, 802 met the inclusion criteria for full text review. Articles were examined using the following objectives: 1) conceptually map the VBHC literature, 2) identify application of Porter’s equation, and 3) identify the methodologies used to measure outcomes, costs, and value. Findings were cross-compared and emergent themes organized to Porter facets of value (outcomes, costs, and value). Our review found the three facets of Porter’s VBHC are established across the literature, however, most studies examine only one or two facets and fail to specify or define all three. Although applied in research, there is a lack of consistency in the actual use of Porter’s definition. Five recommendations for future research using Porter’s VBHC include: (1) pre-selection of outcomes/costs, (2) operationalizing outcomes/costs, (3) value framework creation, (4) data collection, and (5) value calculation.

